# Combinatorial library design for improving isobutanol production in *Saccharomyces cerevisiae*

**DOI:** 10.3389/fbioe.2022.1080024

**Published:** 2022-12-02

**Authors:** Francesca V. Gambacorta, Joshua J. Dietrich, Justin J. Baerwald, Stephanie J. Brown, Yun Su, Brian F. Pfleger

**Affiliations:** ^1^ DOE Great Lakes Bioenergy Research Center, University of Wisconsin-Madison, Madison, WI, United States; ^2^ Department of Chemical and Biological Engineering, University of Wisconsin-Madison, Madison, WI, United States

**Keywords:** yeast, isobutanol, metabolic engineering, combinatorial library, *Saccharomayces cerevisiae*

## Abstract

*Saccharomyces cerevisiae* is the dominant fermentative producer of ethanol in industry and a preferred host for production of other biofuels. That said, rewiring the metabolism of *S. cerevisiae* to produce other fermentation products, such as isobutanol, remains an academic challenge. Many studies report aerobic production of isobutanol, but ethanol remains a substantial by-product under these conditions due to the Crabtree effect. These studies indicate that the native isobutanol pathway is incapable of carrying sufficient flux to displace ethanol. In this report, we screened a combinatorial library of pathway enzymes to identify an isobutanol pathway cassette capable of supporting the growth of a non-ethanol producing *S. cerevisiae*. We began by identifying a diverse set of isobutanol pathway enzyme homologs and combined each open reading frame with varied-strength promoters in a combinatorial, pooled fashion. We applied a growth-coupled screen where a functional isobutanol pathway restored NAD^+^ regeneration during glucose catabolism that is otherwise repressed *via* the Crabtree effect. Using this screen, we isolated a cassette consisting of a mosaic of bacterial and cytosol-localized fungal enzymes that conferred under aerobic conditions the ability to produce 364 mg/L isobutanol (8.8% of the theoretical maximum yield). We next shifted the cofactor usage of the isolated ketol-acid reductoisomerase enzyme in the cassette from NADPH to NADH-preferring to improve redox balance. The approach used herein isolated isobutanol producing strains that approach the best in the literature without producing substantial ethanol titers. Still, the best isolated cassette was insufficient to support anaerobic growth in the absence of ethanol fermentation - indicating the presence of further fundamental gaps in our understanding of yeast fermentation.

## Introduction

**FIGURE 1:**
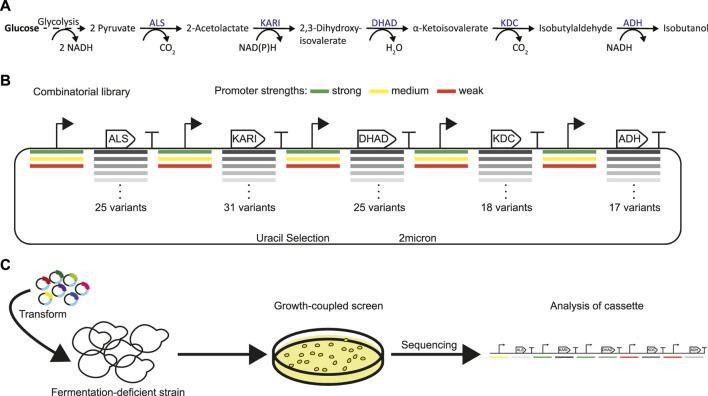
Strategy for identifying a highly active isobutanol pathway cassette. **(A)** Shows the 5 key enzymes responsible for isobutanol production from pyruvate: acetolactate synthase (ALS), ketol-acid reductoisomerase (KARI), dihydroxyacid dehydratase (DHAD), *α*-ketoacid decarboxylase (KDC), and alcohol dehydrogenase (ADH). **(B)** Combinatorial isobutanol pathway library design. There are 25 ALS homologs, 31 KARI homologs, 25 DHAD homologs, 18 KDC homologs, and 17 ADH homologs; each homolog is expressed with either a strong (green), medium (yellow), or weak (red) strength promoter in a high-copy (2*µ*) plasmid containing *URA3* as a selectable marker. **(C)** Shows the growth-coupled screening strategy for identifying highly active isobutanol pathway cassettes. The expression library is transformed into a fermentation-deficient strain (Pdc-null). Then cells that contain a functional isobutanol pathway cassette can grow due to the regeneration of NAD^+^ *via* the ADH and/or the KARI enzyme. High producers can then be sequenced to identify the enzyme homologs and promoters in the cassette.

There is growing demand for more sustainable and environmentally responsible energy carriers, particularly in the transportation sector. Liquid organic molecules remain the preferred transportation fuel because of their high energy density and the ease with which they can be transported. The drawbacks of ethanol, the dominant first-generation biofuel, have been well documented (Peralta-Yahya et al., 2012). Relative to gasoline, ethanol has a significantly lower energy density, is more corrosive, and absorbs more moisture, greatly limiting the extent to which it can be used (Roussos et al., 2019). Ethanol however is a fermentation product that can be efficiently produced from sugars anaerobically by many industrialized microbes. The situation has motivated development of next-generation biofuels that have superior chemical properties and can be synthesized at near maximum theoretical yields. Isobutanol is one such molecule that has been targeted by academic and industrial researchers. Isobutanol is a branched-chain, four-carbon alcohol with a higher energy density and lower hygroscopicity than ethanol (Roussos et al., 2019). Isobutanol can be used as a fuel in internal combustion engines and oligomerized into higher molecular weight hydrocarbons to serve as jet and diesel fuels (Buijs et al., 2013). Isobutanol can be produced biologically from two pyruvate molecules through five enzymatic reactions catalyzed by: acetolactate synthase (ALS), ketol-acid reductoisomerase (KARI), dihydroxyacid dehydratase (DHAD), *α*-ketoacid decarboxylase (KDC), and alcohol dehydrogenase (ADH) (Figure 1A). The yeast *Saccharomyces cerevisiae* natively possesses each of these enzymes, and wild-type strains may produce up to ∼50 mg/L of isobutanol (Longo et al., 1992). This natural ability of *S. cerevisiae* to produce isobutanol, together with its robust genetic toolkit, fast growth rate, and tolerance to a variety of stressors present in industrial fermentations have made it a popular host for metabolic engineering efforts to improve isobutanol production (Gambacorta et al., 2020).

Although there have been numerous efforts to engineer *S. cerevisiae* for isobutanol production, the titers, rates, and yields achieved by academic researchers remain below the threshold for industrial feasibility. To date, the highest *S. cerevisiae* isobutanol yield reported in academic literature is only 14% of the maximum theoretical yield (Wess et al., 2019), far short of the 95% yield estimated for industrial viability (Cai et al., 2018). This low yield is due to the fact that isobutanol production in *S. cerevisiae* is overpowered by native pyruvate-to-ethanol flux. Efforts to eliminate ethanol production by deleting either the complete set of three pyruvate decarboxylase enzymes (*PDC1*, *PDC5*, and *PDC6*) or the set of six alcohol dehydrogenases (*ADH1*, *ADH2*, *ADH3*, *ADH4*, *ADH5*, and *SFA1*) have proven challenging since strains lacking these enzymes experience severe physiological defects (Flikweert et al., 1996; de Smidt et al., 2012). Strains lacking pyruvate decarboxylase cannot synthesize acetyl-CoA in the cytosol and require a C2-compound (e.g. ethanol or acetate) in the medium to grow (Flikweert et al., 1996). Additionally, both PDC and ADH-lacking strains have further growth defects because ethanol production, under both aerobic and anaerobic conditions, plays an essential role in replenishing the NAD^+^ cofactor required for glycolysis and cell growth.

Metabolic modeling suggests that isobutanol can theoretically replace ethanol as a product since it is redox-balanced with glycolysis (i.e. requires two reducing equivalents) (Milne et al., 2016). However, while isobutanol production is redox balanced with glycolysis, its cofactor specificity may not be. Naturally, most KARI’s have a higher specificity towards NADPH, which would result in a NADPH shortage and NADH excess (Brinkmann-Chen et al., 2013). This imbalance can be overcome by supplying a means for converting glycolytic NADH into NADPH, changing the KARI cofactor specificity through protein engineering, or using a naturally NADH-dependent KARI enzyme. In addition to balancing cofactor specificity, the carbon flux through the isobutanol pathway must match the naturally high rate of glycolysis to ensure that the NAD^+^ needed for glycolysis is regenerated quickly enough. A large literature base describing the biochemistry, relative activity, and engineering of each enzyme exists. However, to date, no academic group has reported a combination of enzymes that can enable anaerobic isobutanol fermentation in non-ethanol producing *S. cerevisiae*.

A powerful approach for identifying optimal combinations of enzymes and expression levels is through the building and screening of a combinatorial library of pathway cassettes, in which expression levels and/or homolog identity is varied for each enzyme in the pathway (Jeschek et al., 2017). A screen can then be used to identify strains with improved properties and the DNA cassettes that produce the desired phenotype can be subsequently determined and reverse engineered. In designing a library, it is simplest to keep the homolog identities constant and vary only the expression levels, which can be done by exchanging promoters or other control elements. Alternatively, the specific activity of each enzyme can be varied by using homologs from across the tree of life or proteins engineered/evolved to have altered properties. A combined approach increases library complexity, but it can also streamline engineering efforts by exploring diverse homologs and expression levels simultaneously. That said, to fully leverage a large library, a high-throughput screening process must exist. When screening throughput is high enough relative to the library size, it is possible to explore a library fully and have high confidence that every permutation has been sampled (Zhang et al., 2022). If screening throughput is not sufficient, it may be possible to use machine learning methods on a small subset of the library and predict cassettes that will produce the desired phenotype (Zhou et al., 2018; Zhang et al., 2020; Kumar et al., 2021).

In this study, we used bioinformatic methods to identify a diverse set of isobutanol enzyme variants. We then built a combinatorial pathway library that had diversity in both enzyme homologs and expression levels by combining the bioprospected open reading frames with varying strength promoters. By screening the combinatorial pathway library with a growth-coupled strategy, we identified a higher-flux isobutanol cassette that produced an isobutanol titer of 364 mg/L and a yield of 36 mg isobutanol/g glucose (8.8% of the theoretical maximum yield) under aerobic conditions, while not producing ethanol. In an effort to improve redox-balance, we mutated the KARI enzyme in the best cassette to change the cofactor usage from NADPH to NADH-preferring. Unfortunately, the resulting pathway could neither enable anaerobic growth nor improve isobutanol titers under micro-aerobic conditions (sealed serum vials). Our approach was successful in isolating an improved strain from a small number of tested variants. This success motivates further work to improve the transformation efficiency of Pdc^−^ strains and/or limit the library size such that the ideal combination of pathway enzymes can be found.

## Materials and methods

### Media

When culturing strains for transformation, YPGE medium was used, containing 10 g/L yeast extract, 20 g/L peptone, 3% glycerol, and 2% ethanol. After transformation of the library or individual plasmids, strains were grown on defined synthetic complete media minus uracil (SC-ura), containing 6.7 g/L yeast nitrogen base (YNB) without amino acids with ammonium sulfate, 1.52 g/L drop-out mix synthetic minus uracil and leucine, 380 mg/L leucine, and appropriate carbon sources. SCD-ura medium contained 2% glucose, SCGE-ura medium contained 3% glycerol and 2% ethanol, SCDA-ura medium contained 2% glucose and 70 mM sodium acetate, and SCDE-ura medium contained 2% glucose and 2% ethanol. Solid media also contained 2.5% agar.

### Computational methods

Sequence similarity networks were created for each of the isobutanol enzymes (ALS, KARI, DHAD, KDC, and ADH) using the Enzyme Function Initiative-Enzyme Similarity Tool (EFI-EST) (Zallot et al., 2019) and visualized using Cytoscape (Shannon et al., 2003). The input for EFI-EST were either sequences or protein family: ALS from *Lactobacillus plantarum* (Uniprot ID: A0A0G9FA99), KARI from Pfam families PF01450 and PF07991, DHAD from *S. cerevisiae* (Uniprot ID: P39522), KDC from *S. cerevisiae* (Uniprot ID: Q06408), and ADH from *S. cerevisiae* (Uniprot ID: P38113). A diverse set of variants was chosen from the resulting network by 1) gathering homologs from clusters known to contain active enzyme variants and 2) bioprospecting the network to identify diverse sequences from a variety of kingdoms/phyla (fungi, ascomycota, firmicutes, proteobacteria, and actinobacteria). A simple Hamming distance calculation was used to maximize diversity. To ensure we had a fully cytosolic compartmentalized isobutanol pathway, the sequences were run through Mitoprot (Claros and Vincens, 1996) and any predicted mitochondrial localization sequences were removed. Pairwise amino acid identity between the proteins in each grouping was found using ClustalOmega (Madeira et al., 2022). See Supplementary Data Sheet S1 for sequences and Supplementary Data Sheet S2 for percent identity matrices.

### Library construction

Library construction was performed by the DOE Joint Genome Institute (JGI). In brief, codon-optimized enzyme coding sequences (CDS), promoters, and terminators were first cloned into a pENTR vector to generate the part vectors. Transcription units (promoter-gene-terminator sets) were generated by Golden Gate assembly of the part vectors. The combinatorial library (five gene isobutanol cassettes) was then assembled in a pooled fashion (one-pot) using Gibson Assembly into a high-copy vector, pCC1FOSY, a pCC1FOS-based vector with a 2µ yeast origin and *URA3*-selection (Supplementary Table S1). Specifically the one-pot mixture consisted of 116 ORFs (25 ALS homologs, 31 KARI homologs, 25 DHAD homologs, 18 KDC homologs, and 17 ADH homologs), 15 promoters (3 ALS promoters, 3 KARI promoters, 3 DHAD promoters, 3 KDC promoters, and 3 ADH promoters), and 1 terminator (1 ALS terminator, 1 KARI terminator, 1 DHAD terminator, 1 KDC terminator, and 1 ADH terminator) . This yielded 1.44 billion unique 13 kb isobutanol pathway cassettes (3*25*3*31*3*25*3*18*3*17). The pooled library DNA was sheared to an average size of 2 kb and sequenced on a PacBio Sequel II. Each read was analyzed for the presence of a promoter-gene junction and then counted towards the number of reads for the corresponding promoter-gene pair. Each of the 348 promoter-gene pairs was present, and the largest fold-change difference between promoter-gene pair abundance was 12, while most were within a much narrower distribution. PacBio sequencing also revealed a high library fidelity, with only a small fraction of reads showing incorrect sequences (Supplementary Data Sheet S3).

### Library screening and genotyping

The screening strain was generated by deleting *URA3* in *S. cerevisiae* GG570 (Flikweert et al., 1996) by CRISPR/Cas9. In brief, a sgRNA sequence targeting the CDS was identified by CRISpy-pop (sgRNA = gca​cac​ggt​gtg​gtg​ggc​cc) and cloned into the pXIPHOS (NatMX) plasmid as described previously (Stoneman et al., 2020), resulting in plasmid pXIP-URA3-2 (Supplementary Table S1). The CRISPR/Cas9 plasmid was transformed along with a PCR product repair template of the homology flanking the targeted gene and plated on YPGE with nourseothricin. The gene deletion was confirmed by PCR of gDNA and Sanger-sequencing.

The JGI library was then electroporated into *S. cerevisiae* FVG454, GG570*ura3*Δ, as previously described (Becker and Guarente, 1991) and plated on SCDA-ura. Plates were incubated at 30°C for ∼1 month. An additional transformation of the library was conducted in the same manner, but without plating. Instead, after outgrowth in rich medium, the transformed library was used to inoculate two flasks, each with 1 L of SCDA-ura. The flasks were incubated at 30°C and 250 rpm for 10 days, then a portion was plated on SCDA-ura. Together, these screens only resulted in 28 colonies that could be successfully restreaked. These 28 colonies were then further tested with fermentation experiments in 24-well plates.

For genotyping of library colonies, cells were boiled in 20 mM NaOH for 5 min, and the resulting lysate used as a PCR template for a LongAmp Taq (NEB) reaction spanning the entire isobutanol cassette. The resulting amplicon was verified to be the correct length by agarose gel and then subjected to Sanger or Nanopore sequencing.

### Yeast plasmid isolation

Yeast cultures were grown in SCGE-ura for 4 days, and yeast plasmids were isolated with a Zymoprep™ Yeast Plasmid Miniprep II (Zymo Research), then transformed into TransforMax EPI300 Electrocompetent *Escherichia coli* (Lucigen). The resulting *E. coli* cells were grown overnight in LB with the appropriate antibiotic along with CopyControl™ Induction Solution (Lucigen), and plasmids were miniprepped using a QIAprep Spin Miniprep Kit (Qiagen).

### Fermentations and spot plates

Fermentation experiments were done in either 15 ml culture tubes, 50 ml rubber-stoppered serum vials, or 24-well plates. For the 24-well plate experiments, individual colonies were restreaked onto SCGE-ura plates, then the patch was used to inoculate 1 ml SCDE-ura and grown for 4 days at 30°C and 250 rpm. For culture tube and serum vial experiments, individual colonies were first used to inoculate 5 ml pre-cultures of SCGE-ura and grown aerobically for 3 days at 30°C and 250 rpm. Cultures were then washed twice with sterile water and used to inoculate either 5 ml (culture tubes) or 30 ml (serum vials) of SCD-ura + 70 mM ethanol at an optical density (OD_600_) of 0.1. Culture tubes were then grown for 3 days, and serum vials for 13 days, at 30°C and 250 rpm before analyzing fermentation products. For spot plate experiments, saturated SCGE-ura cultures were diluted to an OD_600_ of 0.1 and 10 µl suspensions were spotted onto agar plates with the appropriate medium. For anaerobic growth, a Coy anaerobic chamber (5% H_2_, 5% CO_2_, and 90% N_2_) was used with stir bars on a magnetic stir plate to prevent flocculation. Medium used in anaerobic experiments was degassed for >12 h prior to use.

### Fermentation product analysis

After fermentations were completed, cultures were placed on ice for 10 min (if serum vials), then centrifuged at 3000 g for 5 min. Supernatant was then removed and used for further analysis. Isobutanol titer was measured *via* headspace sampling of the supernatant and analysis by GC-MS. Specifically, the equipment used included the following: an Agilent 7890A GC system (Agilent Technologies, Inc. Palo Alto, CA); a LPAL3 autosampler and sample preparation system equipped with a heated agitator/stirrer and heated headspace sampling syringe (Agilent Technologies, Inc. Palo Alto, CA); and a Pegasus 4D ToF-MS (Leco Corp., Saint Joseph, Michi- gan). Typical analysis range is 0.065 mM–8.4 mM isobutanol and uses an aliquot volume of 500 ml and 2-Methylpropyl-d9 alcohol as an internal standard. Instrument run control and conditions are set by the Chromatof (®Leco Corp.) software (version 4.72.0.0) provided with the Pegasus 4D GcxGC ToF MS system. Samples were incubated at 70°C for 5 min in the heated agitator set to 350 rpm. 0.5 ml of the headspace is then sampled by the autosampler with a 2.5 ml gastight syringe heated to 75°C and injected into the GC system. The sample was withdrawn at 100 ml/s and injected into the GC at 1,000 ml/s. The syringe was purged with nitrogen gas for 0.5 min prior to the next injection. The analytical capillary GC column was a Stabilwax-DA^®^ (Restek, Inc. Bellefonte, PA) length 30 m, 0.25 mm ID, 0.25 mm film thickness. Helium was used as a carrier gas with a pressure corrected constant flow rate of 1 ml/min. The GC inlet was fitted with a 4 mm deactivated glass liner and held at 250°C throughout the run. The inlet split ratio was set to 50:1. The GC oven was initially set to 50°C and held for 1 min then increased to 200°C at 40°C/min and held at 200°C for 5 min. The filaments of the mass spectrometer were turned on 42.5 s after injection and 10 spectra/sec were recorded from m/z 10 to m/z 250. The MS source temp was 200°C, electron energy set to 70 eV, and the detector voltage was adjusted to approximately 50 V above the minimum voltage determined by the instrument tune check procedure. The peak area of isobutanol was measured using the extracted ion chromatogram of m/z 43 and the peak area of 2-Methylpropyl-d9 alcohol was measured from the extracted ion chromatogram of m/z 46.

End-product analytes (glucose, ethanol, pyruvate) were measured with an analytical system consisting of an Agilent 1260 Infinity HPLC system (Agilent Technologies, Inc., Palo Alto, CA) with a quaternary pump, chilled (4°C) autosampler, vacuum degasser, refractive index detector, and a Aminex HPX-87H column with a Cation-H guard column (BioRad, Inc. Hercules, CA; 300 × 7.8 mm, cat# 125–0140). Operating parameters were as follows: 0.02 N H_2_SO_4_ mobile phase, 0.500 ml/min flow rate, 50°C column temperature, 50°C detector temperature, 28 min run time, and a 50 μl injection volume. Instrument control, data collection and analysis/calculation are done using Chem Station V. B04.03 software (Agilent Technologies, Inc., Palo Alto, CA).

### Protein expression and purification

The wild-type *Lachnospiraceae bacterium* KARI gene (denoted *LbIlvC*) and its variants were cloned into pET-28a (from EMD Biosciences) with a C-terminal 6xHis tag. The pET28-*LbIlvC* (pFVG607), pET28-*LbIlvC* ^DD^ (pFVG608), and pET28-*LbIlvC* ^DDV^ (pFVG609) plasmids were transformed into *E. coli* BL21(DE3) (from New England Biosciences) (Supplementary Table S1). Cultures were grown at 37°C with shaking in LB supplemented with 50 mg/L kanamycin until the OD_600_ reached 0.8. Flasks were cooled in a 16°C bath, induced with 100 *µ*M isopropyl-*β*-d-thiogalactopyranoside (IPTG), and incubated overnight at 21°C. Cells were harvested by centrifugation (8,000 rpm at 4°C for 15 min) and flash frozen as pellets in liquid nitrogen. The frozen cell pellets were broken by sonication (Fisher 550 Sonic Dismembrator, amplitude = 30%, time = 10 min with 1 s on/1 s off) while on ice in Buffer A (20 mM Tris pH 7.4, 30 mM imidazole, 100 mM NaCl, 10 mM MgCl_2_). The lysate was cleared by centrifugation (12,000 rpm at 4°C for 30 min) and filtered with a 0.4 *µ*M filter. The proteins were purified by immobilized metal affinity chromatography (IMAC) over 5 ml Histrap High Performance (HP) columns (Cytiva, Sweden), using an AKTA Start system. All purification steps were performed at 4°C. The proteins were eluted with a linear gradi ent from buffer A to 100% buffer B (20 mM Tris pH 7.4, 500 mM imidazole, 100 mM NaCl, 10 mM MgCl_2_, and 1 mM DTT (added before use). Fractions containing the protein were pooled, buffer exchanged, and concentrated into Buffer C (8 mM Tris pH 7.4, 20 mM NaCl, 1 mM MgCl_2_, and 5 wt% glycerol) using Amicon Ultra centrifugal filters. Aliquots were stored at −80°C and used within 1 month.

### Kinetic assay

*LbIlvC* activities were assayed by monitoring NAD(P)H consumption at 340 nm on a Nanodrop with a 1 cm path length. The assay buffer contained 250 mM potassium phosphate pH 7, 1 mM DTT, 200 μM NADPH or NADH, 10 mM 2-acetolactate, 10 mM MgCl_2_, and 1,500 nM purified enzyme. The concentrations of the purified enzymes were determined using the Nanodrop. An unpaired *t*-test was performed to determine statistical significance.

## Results

### Combinatorial isobutanol pathway library design

The goal of this project was to construct a combinatorial library of genes from which a strain demonstrating a high-flux isobutanol pathway could be isolated. Variability was introduced in the library on two levels: coding sequences (i.e. DNA encoding enzyme variants from a homology search) and enzyme expression (i.e. varied transcription levels from distinct, previously characterized promoters (Lee et al., 2015)). We identified homologs of each enzyme within the isobutanol pathway by the use of bioprospecting. A diverse set of variants were chosen from the EFI-EST (Zallot et al., 2019) sequence similarity map by: 1) gathering homologs from clusters known to contain active enzyme variants, and 2) bioprospecting the network to identify diverse sequences from a variety of kingdom/phyla (fungi, ascomycota, firmicutes, proteobacteria, and actinobacteria). Specifically, 25 ALS homologs (23–62% identity to *S. cerevisiae ILV2*), 31 KARI homologs (22–67% identity to *S. cerevisiae ILV5*), 25 DHAD homologs (39–63% identity to *S. cerevisiae ILV3*), 18 KDC homologs (21–34% identity to *S. cerevisiae ARO10*), and 17 ADH homologs (23–49% identity to *S. cerevisiae AHD5*) were chosen (Figure 1B, Supplementary Figure S1, Supplementary Data Sheet S2). To ensure we had an entirely cytosolic localized isobutanol pathway, all predicted mitochondrial localization sequences from Mitoprot (Claros and Vincens, 1996) were removed. Genes were codon-optimized for *S. cerevisiae*, and each homolog was expressed under control of a well-characterized strong, medium, or weak promoter. The resulting transcriptional units for each gene were then used to build the final pooled plasmid library in which each plasmid contained a gene encoding each of the five required isobutanol pathway enzymes. In total, the library could consist of 1.44 billion unique combinations (Figure 1B). The library was sized to match the typical transformation efficiency of *S. cerevisiae* laboratory strains. A portion of the pooled library was sequenced using PacBio. The resulting reads were aligned to the designed genes and each occurrence of gene, promoter, and promoter-gene pair were counted. All promoters and CDSs in the library were represented and their distribution is detailed in Supplementary Data Sheet S3. Each of the 348 promoter-gene pairs was present, and the largest fold-change difference between promoter-gene pair abundance was 12, while most were within a much narrower distribution. Overall, the sequencing results indicate that the library achieved high diversity and fidelity.

### Growth-coupled library screening

The large library size necessitated the use of a high-throughput screen to find plasmids that generate a high-flux pathway to isobutanol. Lacking a high-throughput analytical pipeline or a eukaryotic biosensor that senses isobutanol directly, we opted for a growth-coupled approach (Figure 1C) where strains possessing a high-flux isobutanol pathway would grow more quickly. At the outset, we envisioned a growth selection (anaerobic) but due to technical challenges, settled for growth enrichment (aerobic). We used a *S. cerevisiae* strain designated GG570 (Flikweert et al., 1996) whose genome lacks each of the three genes encoding pyruvate decarboxylase (T2-3D::*pdc1*Δ, *pdc5*Δ, and *pdc6*Δ). This strain grows slowly on glucose aerobically in part because of the Crabtree effect (de Alteriis et al., 2018) and cannot grow on glucose anaerobically because of the lost ability to regenerate NAD^+^ through ethanol fermentation. We hypothesized that high-flux isobutanol pathways would restore NAD^+^ regeneration by the ADH and KARI (if NADH-dependent) reactions and improve growth rates over the base strain. To facilitate library cloning, we deleted the *URA3* gene from GG570, resulting in strain FVG454 (Table 1), which required a plasmid containing the *URA3* marker for growth on media lacking uracil.

**TABLE 1:** *S. cerevisiae* strains used in this study.

Strain name	Description
GG570 (Flikweert et al., 1996)	T2-3D::*pdc1*Δ, *pdc5*Δ, *pdc6*Δ
FVG454	GG570:: *ura3*Δ
sJD189	FVG454 + e.v. (pCC1FOSY)
sJD107	FVG454 + #2 cassette (p#2)
sJD195	FVG454 + #1 cassette (p#1)
sFVG612	FVG454 + #2 cassette with *LbllvC* ^DD^ (pFVG605)
sFVG613	FVG454 + #2 cassette with *LbllvC* ^DDV^ (pFVG606)

**FIGURE 2:**
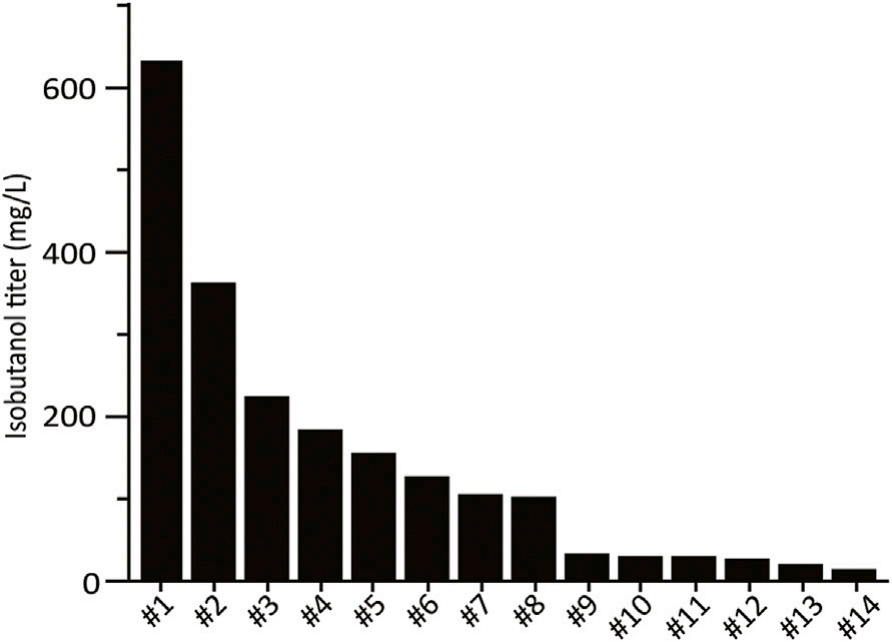
High-throughput growth-coupled screening results. Isobutanol titer from the 28 colonies isolated from the initial screen. Fermentation of one replicate for each colony was performed under aerobic conditions in 24-well plates in SCDE-ura. Isobutanol titer was measured after 4 days. Colonies for which no isobutanol was detected are not shown (14/28).

The combinatorial isobutanol pathway library was transformed into FVG454 *via* electroporation. The resulting cells were plated onto SCDA-ura and allowed to grow anaerobically at 30°C for 1 month. The transformation resulted in 0 colonies and we hypothesized that this growth selection was too strict; under anaerobic conditions, cell growth is completely dependent on isobutanol production for NAD^+^ regeneration as oxidative phosphorylation cannot occur anaerobically. Next, we decided to try a growth enrichment where the combinatorial isobutanol pathway library was transformed into FVG454 *via* electroporation and the resulting cells were plated onto SCDA-ura and allowed to grow aerobically at 30°C for 1 month. Under aerobic conditions, cell growth is dependent on NAD^+^ regeneration through both oxidative phosphorylation and isobutanol production. From the aerobic transformation, only 28 colonies grew from the ∼5 × 10^4^ unique genotypes that were screened (estimated library coverage of 0.0035%). A comprehensive screen was not possible due the limited transformation efficiency (10^3^ cfu/μg) of the screening strain and the large plasmid size of 25.3 kb. The isobutanol production of the twenty-eight colonies was tested by performing a 4-day growth experiment in SCDE-ura medium (Figure 2). Half (14/28) of the colonies produced isobutanol and the best strain achieved an isobutanol titer of 633 mg/L, thereby validating our growth-coupled screening approach. The plasmids containing the isobutanol pathway cassettes were then isolated from the top ten producing strains. Each plasmid was sequenced with Sanger and/or Nanopore sequencing to identify the enzyme homologs and promoter strength of each gene in the cassettes (Table 2).

**TABLE 2:** Homolog and expression level identification of the top 10 isobutanol producers from Figure 2 *via* Sanger and Nanopore sequencing. Green, yellow, and red boxes represent a strong, medium, or weak strength promoter, respectively (See Supplementary Data Sheet S1 for Uniprot IDs).

	ALS		KARI		DHAD		KDC		ADH	
Cassette	Promoter	Homolog	Promoter	Homolog	Promoter	Homolog	Promoter	Homolog	Promoter	Homolog
#1	*P*_*SAC6*_	*Trichoderma gamsii*	*P*_*HHF2*_	*Olsenella scatoligenes*	*P*_*RET2*_	*Hypoxylon sp.*	*P*_*POP6*_	*Dermatophilus congolensis*	*P*_*PAB1*_	*Lactococcus lactis*
#2	*P*_*SAC6*_	*Trichoderma gamsii*	*P*_*HHF2*_	*Lachnospiraceae bacterium*	*P*_*RET2*_	*Jeotgalibaca sp.*	*P*_*ALD6*_	*Frondihabitans sp. 762G35*	*P*_*PAB1*_	*Gluconacetobacter diazotrophicus*
#3	*P*_*SAC6*_	*Talaromyces stipitatus*	*P*_*RNR2*_	*Brevundimonas vesicularis*	*P*_*RPL18B*_	*Saccharomonospora marina*	*P*_*TEF1*_	*Enterococcus rotai*	*P*_*PAB1*_	*Acetobacter indonesiensis*
#4	*P*_*HTB2*_	*Brevibacterium linens*	*P*_*RNR2*_	*Streptomyces griseorubiginosus*	*P*_*RPL18B*_	*Acidimicrobiaceae bacterium*	*P*_*TEF1*_	*Helicobacter ailurogastricus*	*P*_*TEF2*_	*n.a.*
#5	*P*_*SAC6*_	*Bifidobacterium mongoliense*	*P*_*RNR2*_	*Slackia exigua*	*P*_*RPL18B*_	*Arthrobacter alpinus*	*P*_*TEF1*_	*Enterococcus rotai*	*P*_*RNR1*_	*Tanticharoenia sakaeratensis*
#6	*P*_*SAC6*_	*Thiohalomonas denitrificans*	*P*_*HHF2*_	*Lactonifactor longoviformis DSM 17459*	*P*_*RPL18B*_	*Penicillium arizonense*	*P*_*TEF1*_	*Helicobacter ailurogastricus*	*P*_*PAB1*_	*Arthrobacter sp. RC1.1 241*
#7	*P*_*SAC6*_	*Oidiodendron maius*	*P*_*RNR2*_	*Shewanella sp.*	*P*_*RPL18B*_	*Lactococcus piscium*	*P*_*TEF1*_	*Enterococcus rotai*	*P*_*RNR1*_	*Tanticharoenia sakaeratensis*
#8	*P*_*SAC6*_	*Streptomyces viridochromogenes*	*P*_*RNR2*_	*Shewanella sp.*	*P*_*RPL18B*_	*Methanobrevibacter smithii*	*P*_*TEF1*_	*Helicobacter ailurogastricus*	*P*_*PAB1*_	*Chlamydia trachomatis*
#9	*P*_*SAC6*_	*Aspergillus nomius*	*P*_*HHF1*_	*Clostridium populeti*	*P*_*THD3*_	*Methanosphaera stadtmanae*	*P*_*TEF1*_	*Enterococcus rotai*	*P*_*PAB1*_	*Lactococcus lactis subsp. lactis (strain IL1403)*
#10	*P*_*PGK1*_	*Talaromyces stipitatus (strain ATCC 10500)*	*P*_*RNR2*_	*Alphaproteobacteria bacterium*	*P*_*THD3*_	*Thiohalobacter thiocyanaticus*	*P*_*TEF1*_	*Enterococcus rotai*	*P*_*PAB1*_	*Arthrobacter sp. RC1.1 241*

n.a, not available; sequencing reads could not distinguish the homolog.

**FIGURE 3:**
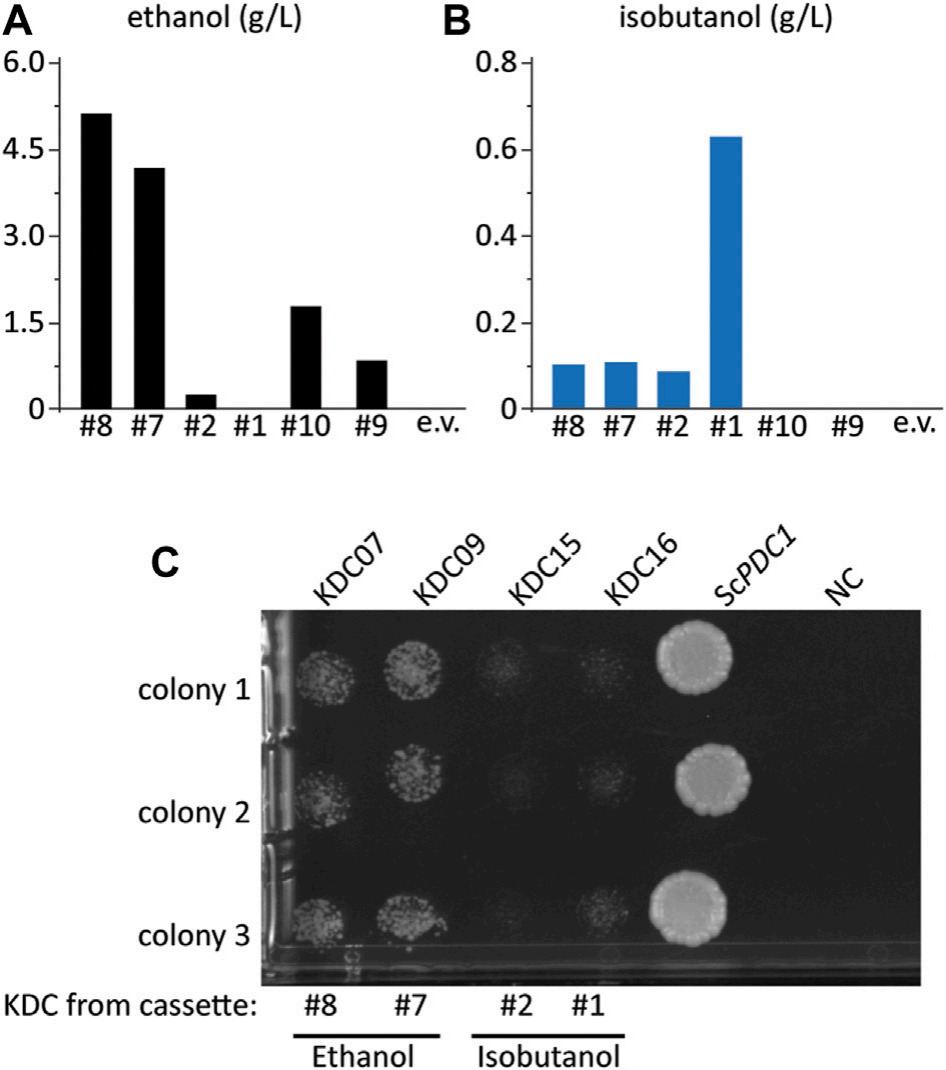
Some KDC enzymes have activity on pyruvate. **(A)** ethanol and **(B)** isobutanol titer from select colonies from the initial screen in Figure 2, FVG454 + isobutanol cassettes (p#8, p#7, p#2, p#1, p#09, p#10 or pCC1FOSY (empty vector)). Fermentation was performed under aerobic conditions with culture tubes in SCDA-ura for 4 days. **(C)** Growth complementation assay to determine which KDC homologs have activity on pyruvate. The KDC homologs from cassettes p#8, p#7, p#2, p#1 and *S. cerevisiae PDC1* (positive control) were cloned into a high copy ura-selectable vector with a strong promoter. All were transformed into the FVG454 strain along with pCC1FOSY (empty vector or NC). 10 μl cell suspensions of 1:100 diluted culture were spot plated on SCD-ura. After 4 days of growth under aerobic conditions, the plates were imaged and the strains containing KDC homologs that have activity on pyruvate grew indicating that NAD^+^ was regenerated *via* ethanol production. Spot plate was done in biological triplicate.

While our initial screening strategy demonstrated the feasibility of the growth-coupled approach to isolate high isobutanol producers, several of the isolates did not produce isobutanol. Instead, we found that many of these strains restored ethanol production, an alternative route to regenerating NAD^+^. Six strains were grown in SCDA-ura medium lacking ethanol (acetate was provided as the C2 donor) so we could accurately measure ethanol production (Figure 3A). Interestingly, of the six cassettes tested, four produced significant amounts of ethanol; specifically, the highest producers were the #8 and #7 cassettes which produced 53 and 42-fold more ethanol than isobutanol, respectively (Figure 3A, Figure 3B). We hypothesized that the KDC homologs in the #8 and #7 cassettes had activity on pyruvate thus allowing the strain to regenerate NAD^+^ *via* ethanol production. To test this hypothesis, we conducted a growth complementation assay by expressing the KDC homologs on a high copy vector and transforming each into the Pdc^−^ strain, FVG454, to observe which could restore growth on medium containing glucose. As expected, the KDC homologs in cassettes #8 (KDC07 from *Helicobacter ailurogastricus*) and #7 (KDC09 from *Enterococcus rotai*) exhibited robust growth, confirming their activity on pyruvate (Figure 3C).

**FIGURE 4:**
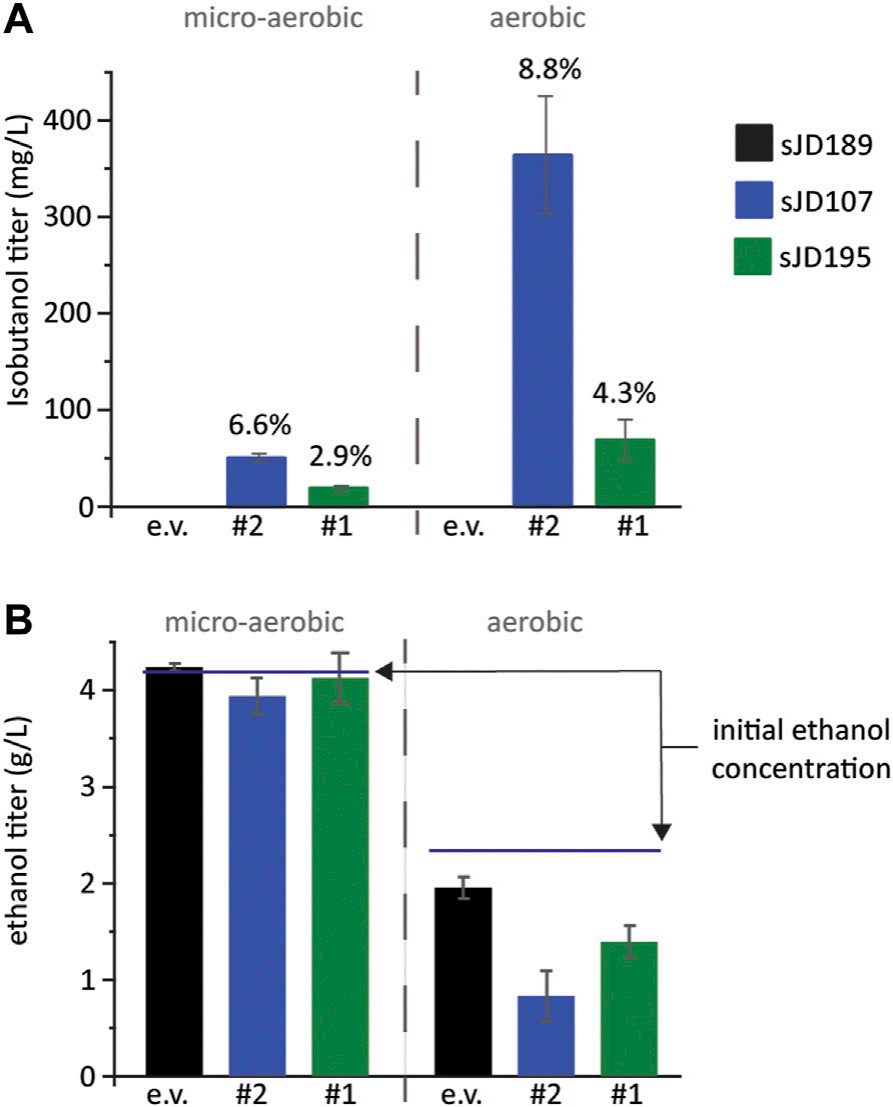
Validation of growth-coupled screening results. **(A)** Isobutanol and **(B)** ethanol titer and from the top two isobutanol-producing cassettes identified. An empty vector, the #2 cassette, and the #1 cassette plasmids were re-transformed into FVG454 resulting in strains sJD189, sJD107, and sJD195, respectively. Fermentations were performed under aerobic and micro-aerobic conditions in SCD-ura + 70 mM ethanol. Aerobic fermentations were conducted in culture tubes for 3 days while micro-aerobic fermentations were conducted in serum vials for 13 days. The percent of maximum theoretical yield achieved is shown above each bar in **(A)** and the starting concentration of ethanol is shown by a line in **(B)**. Error bars represent the standard deviation of the 3 biological replicates.

To validate the isobutanol titers of our top two producing strains, designated #1 and #2, we re-transformed the isolated plasmids back into the base screening strain, FVG454, to create strains sJD195 and sJD107, respectively. The strains were cultivated aerobically (culture tube) and micro-aerobically (serum vial) at 30°C (Figure 4). sJD107 outperformed sJD195 under both micro-aerobic and aerobic conditions. Specifically, the highest producer was sJD107 under aerobic conditions which produced 364 mg/L isobutanol and had a yield of 36 mg isobutanol/g glucose which corresponds to 8.8% of the theoretical maximum yield (Figure 4A). sJD107 also performed well under micro-aerobic conditions where it produced 50 mg/L isobutanol and had a yield of 27.3 mg isobutanol/g glucose which corresponds to 6.6% of the theoretical maximum yield.

To further explore why the #1 and #2 cassettes were so successful (i.e. high isobutanol producers) we took a closer look at the homologs and expression level of the isobutanol pathway enzymes in these cassettes. The KARI promoter and the KDC homolog stood out. The gene dosage for the KARI homolog in both the #1 and #2 cassettes was high (strong promoter), while the weaker isobutanol pathway cassettes (#3, #4, #5, #7, #8, #9, and #10) had a low gene dosage (weak/medium promoter) (Table 2). The strong promoter being required for KARI in our top two producers suggests that KARI is a limiting enzyme in the isobutanol pathway; this result is in agreement with previous studies in *E. coli* where KARI required the highest level of enzyme expression (Ghosh et al., 2019). Additionally, the KDC homologs in the #2 (KDC15 from *Frondihabitans sp. 762G35*) and #1 (KDC16 from *Dermatophilus congolenis*) cassettes were characterized as having minimal activity on pyruvate (Figure 3C); this is as expected since sJD107 and sJD195 produced no ethanol during fermentation in Figure 4B. Taken together, both the isobutanol pathway homologs and promoter strength are important factors for production.

### Anaerobic growth unachievable after balancing NADH/NAD^+^ between glycolysis and isobutanol production

**FIGURE 5:**
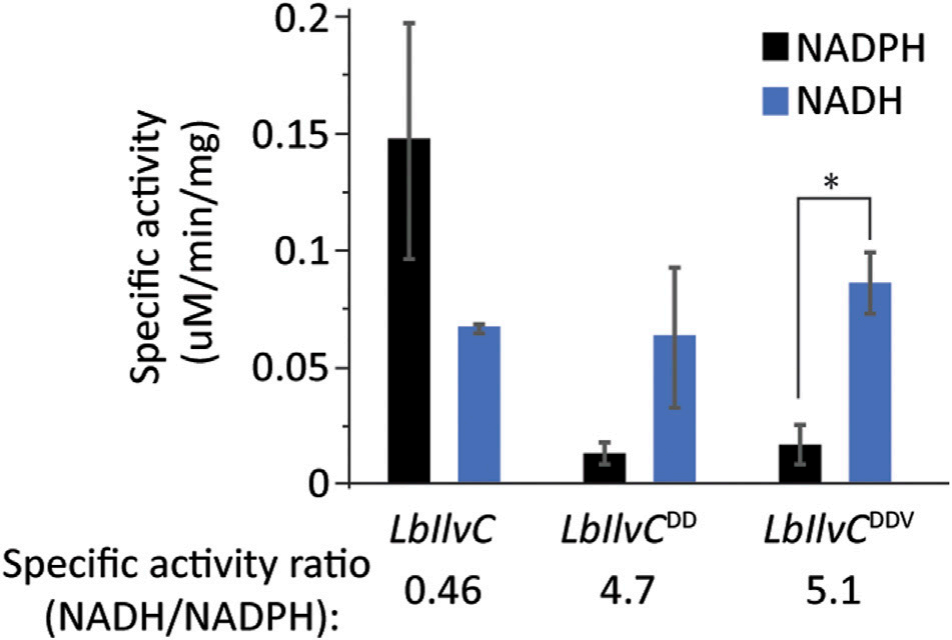
Characterization of *LbIlvC* variants. Specific activities of *LbIlvC* variants using NADPH or NADH, with 2-acetolactate as substrate in excess. All enzymes were purified prior to characterization. Each value represents the average of three independent measurements. The enzyme activities were determined in 250 mM potassium phosphate pH 7 with 1 mM DTT, 200 mM NADPH or NADH, 10 mM 2-acetolactate, and 10 mM MgCl_2_. The concentrations of the purified enzymes were determined using a Nanodrop. The specific activity ratios of *LbIlvC* variants for NADH-to-NADPH are shown under the corresponding bars. Asterisks denote statistically significant differences by pairwise *t*-test (*, *p* < 0.05).

We next aimed to test our best isobutanol producer, sJD107, under industrially relevant anaerobic conditions. Under these conditions, cell growth is completely dependent on isobutanol production for NAD^+^ regeneration as oxidative phosphorylation cannot occur anaerobically. We hypothesized that the capacity of the strain to replenish NAD^+^ by the isobutanol pathway may be limited by a redox cofactor imbalance between the engineered pathway and glycolysis; this is a result of the KARI enzyme from our #2 cassette (*Lachnospiraceae bacterium* homolog) being an NADPH-dependent enzyme. We set out to switch the cofactor preference of the *L. bacterium* KARI enzyme (*LbIlvC*) from being NADPH-dependent to NADH-dependent. The specific residues required for this switch have been previously determined and this strategy was successful in increasing isobutanol production of a different engineered isobutanol pathway in *E. coli* (Bastian et al., 2011; Brinkmann-Chen et al., 2013). We generated two KARI variants, *LbIlvC* ^DD^ (Ser53Asp, Ser55Asp) and *LbIlvC* ^DDV^ (Ser53Asp, Ser55Asp, Ile87Val) and confirmed our variants exhibited a changed cofactor preference (from NADPH to NADH) by purifying the proteins and measuring the specific activity with either NADH or NADPH (Figure 5). Specifically, the *in vitro* assay consisted of monitoring the consumption of NAD(P)H over time at 340 nm when the substrate, 2-acetolactate, was in excess. The *LbIlvC* ^DD^ and *LbIlvC* ^DDV^ variant exhibited a ratio of NADH/NADPH activity (in U/mg) of 4.7 and 5.1 (*p* < 0.05), which is 10.3 or 11.2-fold higher than that for *LbIlvC* variant, respectively (Figure 5).

Once we validated the *LbIlvC* ^DD^ and *LbIlvC* ^DDV^ variants cofactor preference was switched, we cloned them into the #2 cassette in place of the original *LbIlvC* and transformed them into the FVG454 strain resulting in strains sFVG612 and sFVG613, respectively. Neither strain was able to grow anaerobically, indicating that the isobutanol pathway cassette does not support sufficient flux to rapidly replenish the NAD^+^ equivalents needed for glycolysis (i.e. isobutanol production is too low to meet the cellular maintenance energy requirement).

We next asked if the *LbIlvC* ^DD^ and *LbIlvC* ^DDV^ variants would perform better under aerobic conditions. We aerobically cultured strains sJD189, sJD107, sFVG612, and sFVG613 for 3 days in SCD-ura + 70 mM ethanol. Strains sFVG612 and sFVG613, containing either of the NADH-dependent KARI variants, *LbIlvC* ^DD^ and *LbIlvC* ^DDV^, did not outperform sJD107 with the wild-type, NADPH-dependent *LbIlvC*. Strain sJD107 produced ∼280 mg/L isobutanol, which is 4.0 and 1.8-fold higher than the sFVG612 and sFVG613, respectively (Table 3). We suspect the lower isobutanol titer with the *LbIlvC* ^DD^ and *LbIlvC* ^DDV^ variants was due to a reduced specific activity of the KARI enzyme; the *LbIlvC* ^DD^ and *LbIlvC* ^DDV^ variants exhibited a ∼2-fold reduction in catalytic activity (catalytic efficiency of using NADH) relative to *LbIlvC* when using NADPH (Figure 5). While sJD107 produced the highest isobutanol titer, that strain, along with all the other engineered strains, excreted significant amounts of pyruvate (0.7–1.6 g/L) indicating overflow metabolism at the pyruvate node (Table 3). Additional work is needed to enhance flux through the isobutanol pathway to prevent overflow metabolism and allow for the rapid regeneration of NAD^+^ equivalents to develop a truly anaerobic isobutanol fermentative pathway for *S. cerevisiae*.

**TABLE 3:** Fermentation products of *LbIlvC* variants. Aerobic production of strains sJD189, sJD107, sFVG612, and sFVG613. Fermentations were performed in SCD-ura + 70 mM ethanol. Isobutanol, pyruvate and ethanol titers were measured after 3 days. Standard deviation was calculated from 3 biological replicates.

Strain	Plasmid	Isobutanol (mg/L)	Pyruvate (g/L)	Ethanol (g/L)
sJD189	pCC1FOSY	0 ± 0	1.6 ± 0.2	0.3 ± 0.3
sJD107	p#2 (WT *LbIlvC*)	283.6 ± 14.8	0.7 ± 0.1	0 ± 0
sFVG612	pFVG605 (*LbIlvC* ^DD^)	69.3 ± 10	0.9 ± 0	0 ± 0
sFVG613	pFVG606 (*LbIlvC* ^DDV^)	154.2 ± 11.5	1.6 ± 0.2	0 ± 0

## Discussion

To date, isobutanol production from a Pdc^−^ *S. cerevisiae* strain under anaerobic conditions has not been reported in academic literature. We hypothesize this is due to the strain’s limited capacity to replenish NAD^+^ through alternative fermentation pathways like isobutanol production. Here, we used a combinatorial library design and a growth-coupled screen to identify an isobutanol pathway cassette capable of supporting a higher carbon flux. Strain sJD107 which harbored our best cassette, #2, produced 364 mg/L isobutanol and had a yield of 36 mg isobutanol/g glucose under aerobic conditions which corresponds to 8.8% of the theoretical maximum yield. The #2 cassette benefited from having a KDC enzyme with minimal activity on pyruvate which minimized byproduct (ethanol) production. We then aimed to redox cofactor-balance the #2 cassette with glycolysis by changing the cofactor preference of the KARI enzyme in the #2 cassette from NADPH to NADH-preferring. However, the resulting pathway still could neither support anaerobic growth nor improve isobutanol production under aerobic conditions. While we were unable to achieve anaerobic growth, our yields micro-aerobically (serum vial cultivation) exceed previous studies; our sJD107 strain harboring the #2 cassette, achieved a yield of 27.3 mg isobutanol/g glucose which is ∼3.7-fold higher than Milne et al. (Milne et al., 2016) who achieved a yield of 7.4 mg isobutanol/g glucose with a Pdc^−^ strain harboring a cytosolic localized pathway under micro-aerobic conditions. We hypothesize that the capacity of the isobutanol pathway must be further enhanced to achieve anaerobic growth.

The work performed here leaves several opportunities for further improvement. First, it should be noted that our screening method can select for both high isobutanol and high ethanol producing strains so additional screening methods are required to eliminate the false-positive hits or extra care must be taken when selecting KDC homologs. Additionally, the library approach would benefit from enhanced transformation efficiency in Pdc^−^ strains such that large libraries can be screened or working with a reduced library size so libraries could be screened in full coverage.

## Data Availability

The original contributions presented in the study are included in the article/Supplementary Material, further inquiries can be directed to the corresponding author.
