# Supplementary material for: Combinatorial library design for improving isobutanol production in Saccharomyces cerevisiae
Source: Front Bioeng Biotechnol. 2022 Dec 2;10:1080024. doi: 10.3389/fbioe.2022.1080024 (PMC9755324; doi:10.3389/fbioe.2022.1080024)
Supplement: Supplementary file 1 [file Table1.docx]

**Table S1.** Plasmids used in this study

| **Plasmid Name(s)** | **Description** | **Source** |
| --- | --- | --- |
| pCC1FOSY | pCC1FOS_ApraR_*URA3*_2μ | This study |
| pXIP-URA3-2 | pXIPHOS with sgRNA targeting *URA3* | This study |
| p#1-p#28 | pCC1FOSY with various isobutanol pathways inserted | This study |
| pFVG605 | pCC1FOSY_#2 cassette with *LbIlvC*^DD^ instead of WT *LbIlvc* | This study |
| pFVG606 | pCC1FOSY_#2 cassette with *LbIlvC*^DDV^ instead of WT *LbIlvc* | This study |
| pFVG607 | pET-28a_*LbIlvC* | This study |
| pFVG608 | pET-28a_*LbIlvC*^DD^ | This study |
| pFVG609 | pET-28a_*LbIlvC*^DDV^ | This study |
