# Supplementary material for: Combinatorial library design for improving isobutanol production in Saccharomyces cerevisiae
Source: Front Bioeng Biotechnol. 2022 Dec 2;10:1080024. doi: 10.3389/fbioe.2022.1080024 (PMC9755324; doi:10.3389/fbioe.2022.1080024)
Supplement: Supplementary file 3 [file Image1.pdf]

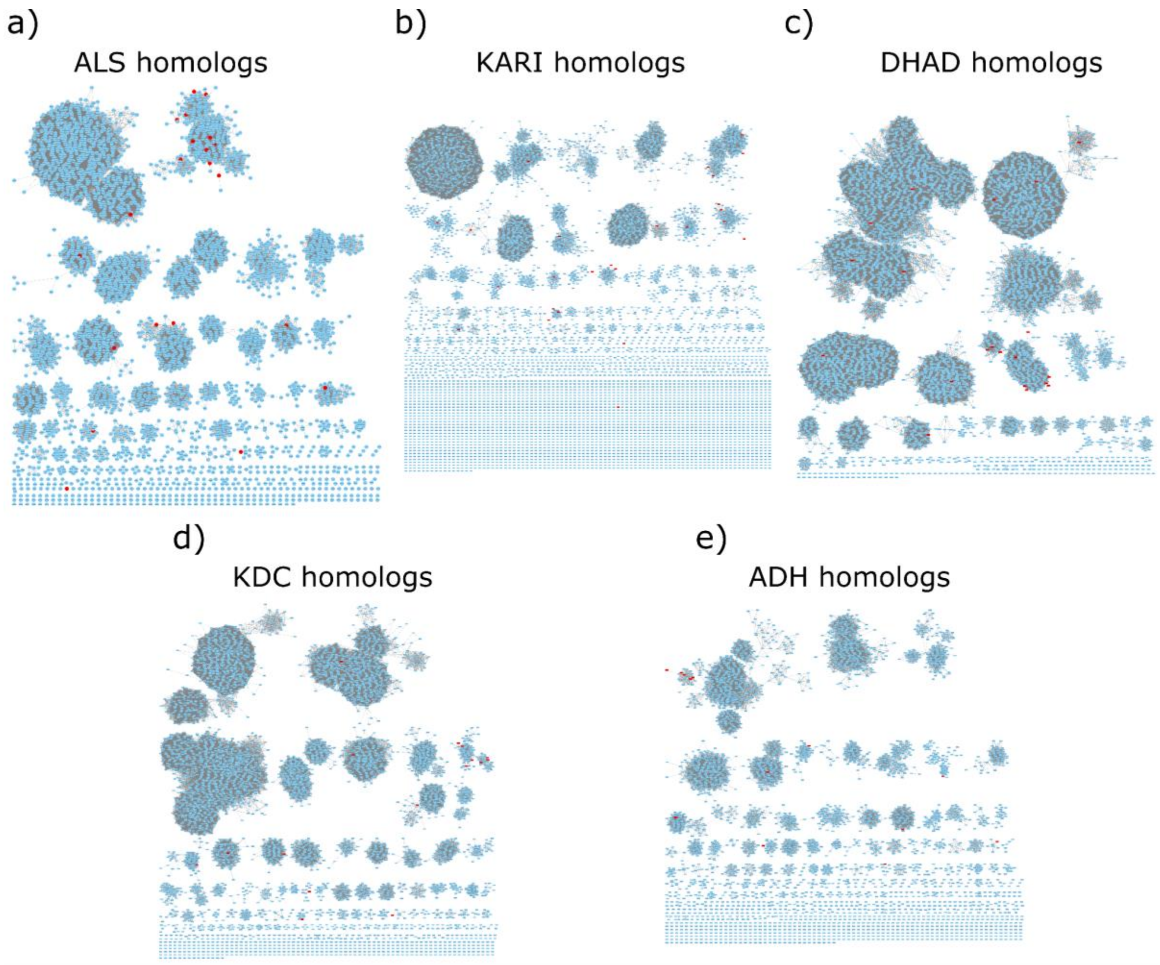

Figure S1. Sequence similarity networks of enzyme homologs visualized with Cytoscape (Shannon et al., 2003) **a)** ALS; **b**) KARI; **c)** DHAD; **d**) KDC; and **e)** ADH homologs. Red nodes denote selected homologs.
